# Association of Gut Microbiota with Age-Related Macular Degeneration and Glaucoma: A Bidirectional Mendelian Randomization Study

**DOI:** 10.3390/nu15214646

**Published:** 2023-11-02

**Authors:** Chen Li, Peirong Lu

**Affiliations:** The First Affiliated Hospital of Soochow University, Pinghai Road 899, Suzhou 215005, China; lceye0902@126.com

**Keywords:** age-related macular degeneration, glaucoma, gut microbiota, mendelian randomization, gut-retina axis

## Abstract

The objective of this study was to examine the correlation between gut microbiota and both age-related macular degeneration (AMD) and glaucoma. Mendelian randomization studies were conducted utilizing the data sourced from the genome-wide association study (GWAS) database for the gut microbiome, AMD, and glaucoma. Single nucleotide polymorphism (SNP) estimates were summarized through five Mendelian randomization (MR) methods. We utilized Cochran’s Q statistic to evaluate the heterogeneity of the instrumental variables (IVs). Additionally, we employed a “leave-one-out” approach to verify the stability of our findings. Inverse variance weighted (IVW) suggests that Eubacterium (oxidoreducens group) and Parabacteroides had a protective effect on AMD. Both weighted median and IVW suggest that Lachnospiraceae (NK4A136 group) and Ruminococcaceae (UCG009) had a protective effect on AMD. However, both weighted median and IVW suggest that Dorea had a risk effect on AMD. Similarly, The IVW of Eubacterium (ventriosum group) showed a risk effect on AMD. The weighted median of Eubacterium (nodatum group), Lachnospiraceae (NC2004 group), and Roseburia had a risk effect on glaucoma. IVW suggested that Ruminococcaceae (UCG004) had a risk effect on glaucoma. Reverse MR analysis found a causal link between Eubacterium (nodatum group) and glaucoma. No causal relationships were found between AMD or glaucoma and the other mentioned bacterial groups. No significant heterogeneity or evidence of horizontal pleiotropy was detected. This study found that certain gut bacteria had protective effects on AMD, while others may be risk factors for AMD or glaucoma. Likewise, reverse MR found that glaucoma led to an increased abundance of certain gut bacteria. Further trials are needed to clarify the specific mechanisms involved.

## 1. Introduction

Age-related macular degeneration (AMD) and glaucoma are prevalent eye diseases that can lead to blindness on a global scale [1,2]. AMD, characterized by the gradual loss of central vision due to progressive damage to the macula, is a primary cause of blindness in the elderly. Clinically, AMD is divided into dry and wet types according to the nature of the lesion. Dry AMD is marked by deterioration of the macula, causing blurred or reduced central vision. Wet AMD is a more severe form of the disease, characterized by the growth of abnormal blood vessels under the macula, leading to rapid and severe vision loss. The development of AMD involves a complex interplay of genetic polymorphism, immune, metabolic, light damage, nutrition, and other factors. During the progression of AMD, microglia and macrophages migrate to the subretinal and choroidal regions, causing a local imbalance in the immune microenvironment of the retinal pigment epithelial cell layer, but the exact mechanism is still unclear [3]. For wet AMD, intraocular injection of anti-VEGF drugs is commonly used for treatment. However, dry AMD, which constitutes 80% of all AMD cases, currently lacks an effective treatment. Glaucoma is a progressive neurodegenerative disorder that is characterized by the gradual deterioration of retinal ganglion cells and their axons [4]. The pathogenesis of glaucoma is still under investigation. Although lowering intraocular pressure (IOP) is the key treatment method, it is difficult to prevent the progression of glaucoma. Current treatment strategies for both AMD and glaucoma still have significant limitations.

The gut microbiota, a diverse microbial community residing in the human intestine, plays a pivotal role in host metabolism, immune defense, and immune tolerance. The concept of the gut-eye axis, first introduced by Vujkovic-Cvijin et al. [5], highlights the significance of the gut microbiota in the development of various eye diseases, such as dry eye syndrome, glaucoma, AMD, uveitis, and diabetic retinopathy (DR). Zinkernagel et al. [6] conducted a study in which they sequenced the gut microbiota of both patients with wet AMD and a control group. The results revealed substantial variations in the abundance of specific gut microbiota between the two groups. Another clinical study on AMD analyzed the Kyoto Encyclopedia of Genes and Genomes (KEGG) database and found 72 metabolic pathways with notable variations in gut microbiota between individuals with AMD and those in the control group, revealing the pathogenesis of gut microbiota involvement in AMD [7]. Gong et al. [8] sequenced the bacterial genomes in fecal samples of patients with primary open-angle glaucoma (POAG) using 16S rRNA V4 gene sequencing and found that the abundance of Prevotella and Escherichia coli was notably higher in POAG patients than in healthy individuals. They also discovered that the development of angle-closure glaucoma is influenced by the gut microbiota. In a comparison of the distribution of gut microbiota between patients with POAG and primary angle-closure glaucoma (PACG), distinct differences were observed [9]. However, most previous studies have struggled to confirm exposure duration and outcomes. Moreover, the correlation between gut microbiota and AMD or glaucoma may be influenced by various confounding factors, including the environment, age, lifestyle, and dietary habits. Therefore, the causal relationship between gut microbiota and both AMD and glaucoma is limited by these confounding factors.

Mendelian randomization (MR) is an analytical technique that utilizes genetic variability as a randomized tool to investigate the exposure of interest, providing valuable insights into the establishment of causality [10]. This innovative approach offers an opportunity to explore the potential causal connections between the gut microbiota and the incidence of AMD or glaucoma. Given that the transmission of genotypes from parents to offspring occurs randomly, the association between genetic variation and outcome remains unaffected by common confounding factors, thus rendering the causal pathway biologically plausible [11]. The application of MR has been widely employed to establish the causal links between the gut microbiota and ocular diseases [12,13,14]. Genome-Wide Association Studies (GWAS) represent a research technique used to identify associations between specific genetic variations and particular diseases across the entire genome. This approach is instrumental in facilitating a greater understanding of the genetic foundations of diseases and uncovering potential targets for treatment. In the current study, the MR analysis was performed using GWAS data, aiming to estimate the causal relationships between the gut microbiota and both AMD and glaucoma.

## 2. Materials and Methods

### 2.1. Data Sources

#### 2.1.1. Gut Microbiota

Genetic variations for gut microbiota are available from the MRC Integrative Epidemiology Unit (IEU) Open GWAS database (http://gwas.mrcieu.ac.uk). The research involved 18,340 participants from 24 cohorts, primarily of European descent (*n* = 13,266). The study employed direct taxonomic binning to screen and categorize microbiota composition based on variable regions V4, V3–V4, and V1–V2 of the 16S rRNA gene. The researchers performed microbiota quantitative trait loci (mbQTL) mapping analysis in order to detect genetic variations in the host and determine their location on genetic loci linked to the abundance levels of bacterial taxa in the gut microbiota. The study identified a total of 131 genera with an average abundance greater than 1% at the lowest taxonomic level, including 12 unknown genera [15]. Therefore, the analysis included 119 genus-level taxonomic units.

#### 2.1.2. AMD

Genetic variations of AMD are also available from the IEU Open GWAS database. A total of 105,248 individuals from 11 different data sources, such as the International AMD Genomics Consortium (IAMDGC) and UK Biobank (UKBB), were involved in the research. The participants were of European descent and consisted of 14,034 cases and 91,214 controls. The study utilized both GWAS and a candidate approach based on 14 early AMD variants to identify early AMD loci [16]. This study merged significant genome-wide mutations (*p* < 5 × 10^−8^) into independent loci. The genes that overlapped with the specified loci were utilized for further biological investigation. In addition, this study also used GCTA for approximate conditional analysis based on meta-analysis to find independent secondary signals in new AMD loci.

#### 2.1.3. Glaucoma

Genetic variations of glaucoma are also obtained from the IEU Open GWAS database. The study performed GWAS on glaucoma and its key endophenotypes (including vertical cup-disc ratio and intraocular pressure) and included 351,696 individuals, these participants were of European descent, including 133,492 cases and 90,939 controls [17]. This study first conducted a GWAS analysis on glaucoma and its main phenotypes, including vertical cup-disc ratio (VCDR) and intraocular pressure. The data was combined through multiple trait analysis of GWAS (MTAG) to identify new loci. The reliability of the new loci was validated by two independent POAG cohorts. In addition, this study created a polygenic risk score (PRS) based on MTAG summary data and further confirmed its clinical significance in early and late glaucoma cohorts.

#### 2.1.4. Instrumental Variables (IVs)

Ivs were selected based on the following selection criteria: (1) For the forward MR analysis, potential Ivs for each genus were identified as single nucleotide polymorphisms (SNPs) within the locus range, with a significant threshold of *p* < 5.0 × 10^−6^. For the reversed MR analysis, SNPs associated with each genus were selected as potential Ivs at a significant threshold (*p* < 5.0 × 10^−8^) within the locus range. (2) In order to determine the linkage disequilibrium (LD) between SNPs, the reference panel used was the European sample data from the 1000 Genomes project. SNPs with an R^2^ < 0.001 and a cluster window size of 10,000 kb were retained, with only the SNP having the smallest *p*-value. (3) Remove palindromic SNPs with intermediate allele frequencies.

### 2.2. Statistical Analysis

**Figure 1:**
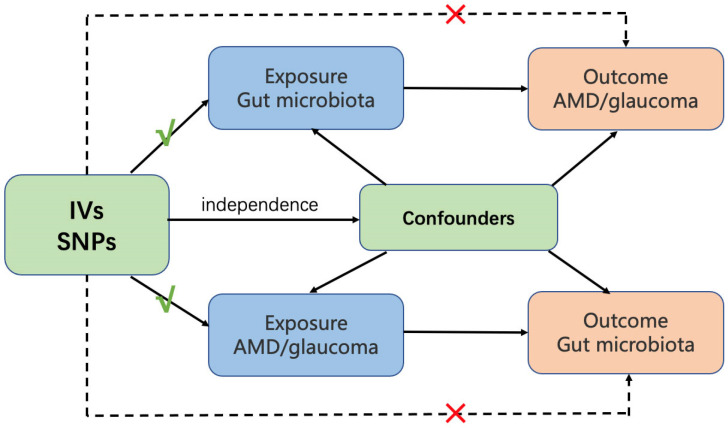
Overview of MR analysis process and major assumptions. AMD, age-related macular degeneration. Ivs, instrumental variables. SNPs, single nucleotide polymorphisms.

This study used five methods, including MR Egger, weighted median, inverse variance weighted (IVW), simple mode, and weighted mode to test the causal relationship between gut microbiota and AMD and glaucoma. The MR-Egger method is one of the commonly used randomization patterns in Mendelian randomization, which evaluates the impact of a factor on a disease based on a linear regression model. Egger regression is used to estimate bias and correct the results, resulting in more accurate causal estimates [18]. The Weighted median method is mainly used to handle biased samples, effectively reducing sample bias and improving the reliability and accuracy of randomized experiments [19]. The IVW merges the Wald estimates of each SNP using meta-analysis to provide a comprehensive estimation of the influence of gut microbiota on AMD and glaucoma. Its advantage is that it can simultaneously consider the effects of multiple genotypes on the study factor, thereby improving the accuracy of causal inference. The IVW result will be unbiased if there is no horizontal pleiotropy [20]. Both the simple mode and weighted mode are frequently implemented randomization patterns that eliminate interfering factors in experimental results by randomly grouping [21]. In this study, Cochran’s IVW Q statistic was utilized to quantify the heterogeneity of Ivs. The MR-PRESSO analysis was employed to identify and mitigate the effects of horizontal pleiotropy by eliminating notable outliers. Furthermore, we conducted the “leave-one-out” analysis to detect potential heterogeneous SNPs by sequentially excluding each instrumental SNP. To evaluate the potential causal relationship between gut microbiota and both AMD and glaucoma, a reverse MR analysis of the two eye diseases with gut microbiota was performed. The method is consistent with the forward MR, and the threshold for significant gene locus selection is *p* < 5 × 10^−8^. All statistical analyses were performed using R version 4.2.2. The MR analyses were conducted using TwosampleMR [22] and MR-PRESSO [23]. The flowchart is presented in Figure 1.

## 3. Results

**Figure 2:**
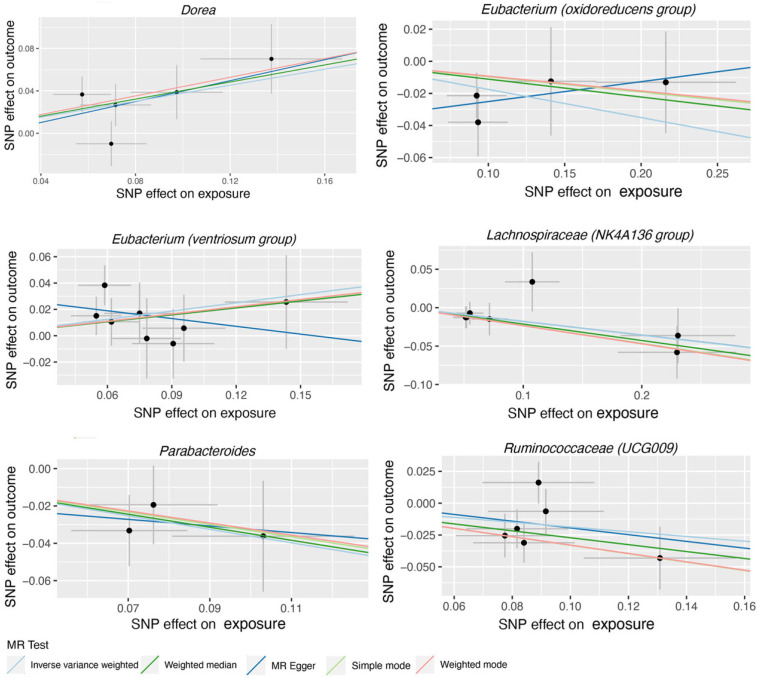
Scatter plots for the casual association between gut microbiota (Dorea, Eubacterium (oxidoreducens group), Eubacterium (ventriosum group), Lachnospiraceae (NK4A136 group), Parabacteroides and Ruminococcaceae (UCG009), and AMD. Each point in the scatter plot represents an SNP. The effect of the same SNP on exposure is placed on the horizontal axis, and the effect on outcome is placed on the vertical axis. The vertical and horizontal lines show the 95% confidence interval (CI) for each SNP. At this point, the slope of the solid line in the plot is each MR estimate. The light blue, light green, dark blue, green, and pink lines correspond to the Inverse Variance Weighted, Simple Mode, MR-Egger, Weighted Median, and Weighted Model methods, respectively. AMD, age-related macular degeneration. SNP, single nucleotide polymorphism.

**Figure 3:**
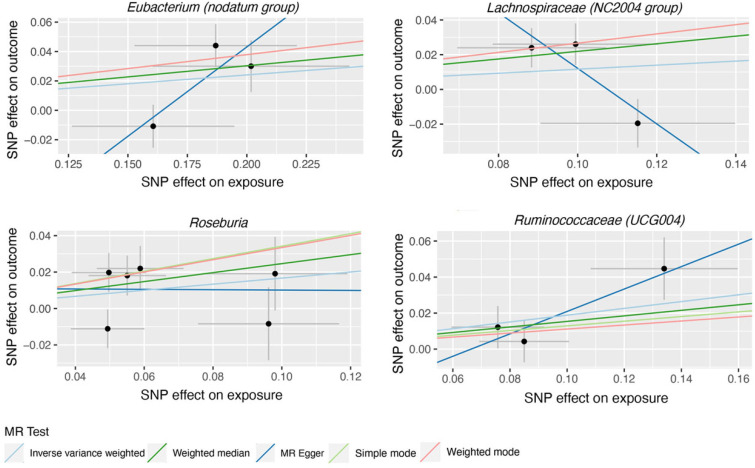
Scatter plots for the casual association between gut microbiota (Eubacterium (nodatum group), Lachnospiraceae (NC2004 group), Roseburia and Ruminococcaceae (UCG004)) and glaucoma. Each point in the scatter plot represents an SNP. The effect of the same SNP on exposure is placed on the horizontal axis, and the effect on outcome is placed on the vertical axis. The vertical and horizontal lines show the 95% CI for each SNP. At this point, the slope of the solid line in the plot is each MR estimate. The light blue, light green, dark blue, green, and pink lines correspond to the Inverse Variance Weighted, Simple Mode, MR-Egger, Weighted Median, and Weighted Model methods, respectively. SNP, single nucleotide polymorphism.

119 bacterial genera were analyzed using 774 SNPs as Ivs based on the specified selection criteria. Detailed information about the selected Ivs is provided in Tables S1 and S2. Tables S3 and S4 present the MR estimates of the impact of bacteria on AMD and glaucoma, respectively. Table 1 outlines the five MR methods used to investigate the relationship between gut microbiota and AMD, while Table 2 exhibits the five MR methods applied to explore the connection between six gut microbiota and glaucoma. Figure 2 illustrates scatter plots depicting the causal associations between gut microbiota (specifically, Dorea, Eubacterium (oxidoreducens group), Eubacterium (ventriosum group), Lachnospiraceae (NK4A136 group), Parabacteroides and Ruminococcaceae (UCG009)) and AMD. Similarly, Figure 3 displayed scatter plots illustrating the casual relationship between gut microbiota and glaucoma, focusing on specific bacterial genera such as Eubacterium (nodatum group), Lachnospiraceae (NC2004 group), Roseburia and Ruminococcaceae (UCG004). The IVW estimate indicates that Eubacterium (oxidoreducens group) (OR = 0.84, 95% CI, 0.70–1.00, *p* = 0.049) and Parabacteroides (OR = 0.70, 95% CI, 0.51–0.96, *p* = 0.025) may have a protective effect against AMD. Similarly, both the weighted median and IVW estimates suggest that Lachnospiraceae (NK4A136 group) (weighted median OR = 0.81, 95% CI, 0.66–0.99, *p* = 0.041; IVW OR = 0.84, 95% CI, 0.71–0.98, *p* = 0.031) and Ruminococcaceae (UCG009) (weighted median OR = 0.76, 95% CI, 0.62–0.94, *p* = 0.011; IVW OR = 0.83, 95% CI, 0.70–0.99, *p* = 0.036) may also provide protection against AMD. Conversely, both the weighted median and IVW estimates suggest that Dorea may increase the risk of AMD (weighted median OR = 1.50, 95% CI, 1.08–2.08, *p* = 0.02; IVW OR = 1.46, 95% CI, 1.15–1.85, *p* = 0.002). The IVW estimate also indicates that Eubacterium (ventriosum group) (OR = 1.23, 95% CI, 1.01–1.50, *p* = 0.038) may increase the risk of AMD. In terms of glaucoma, the weighted median estimates suggest that Eubacterium (nodatum group) (OR = 1.16, 95% CI, 1.01–1.35, *p* = 0.041), Lachnospiraceae (NC2004 group) (OR = 1.24, 95% CI, 1.03–1.51, *p* = 0.026), and Roseburia (OR = 1.28, 95% CI, 1.03–1.59, *p* = 0.028) may increase the risk. The IVW estimate also suggests that Ruminococcaceae (UCG004) may increase the risk of glaucoma (OR = 1.21, 95% CI, 1.02–1.43, *p* = 0.029).

**Figure 4:**
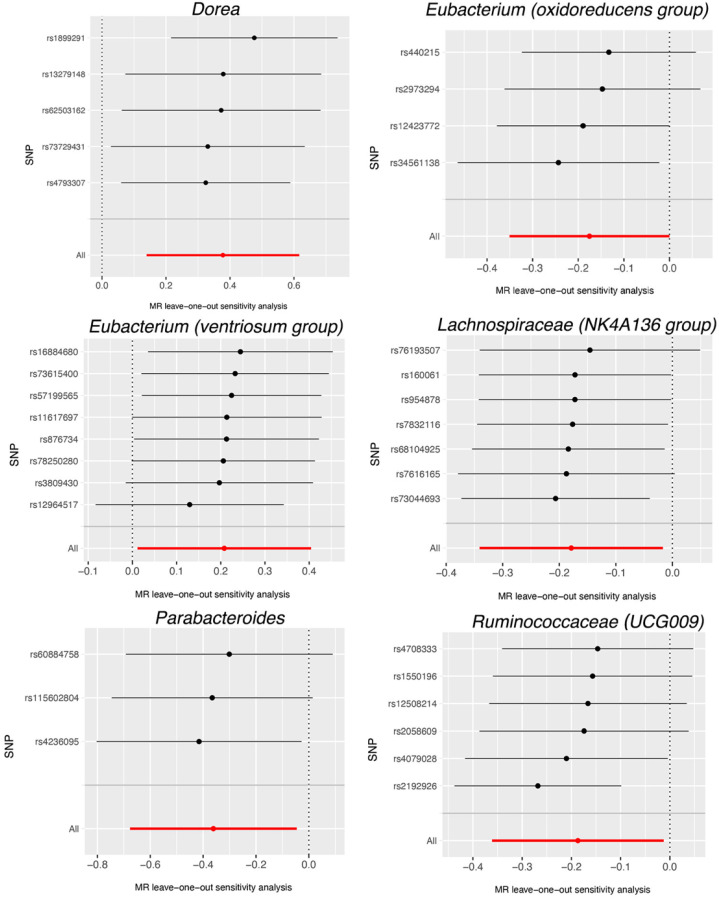
Leave-one-out plots for the causal association between gut microbiota (Dorea, Eubacterium (oxidoreducens group), Eubacterium (ventriosum group), Lachnospiraceae (NK4A136 group), Parabacteroides and Ruminococcaceae (UCG009), and AMD. The leave-one-out plot presents how the causal estimates (point with horizontal circle) for the effect of gut on AMD were influenced by the removal of a single variant. The bars indicate the confidence interval of MR estimates. AMD, age-related macular degeneration. SNP, single nucleotide polymorphism.

**Figure 5:**
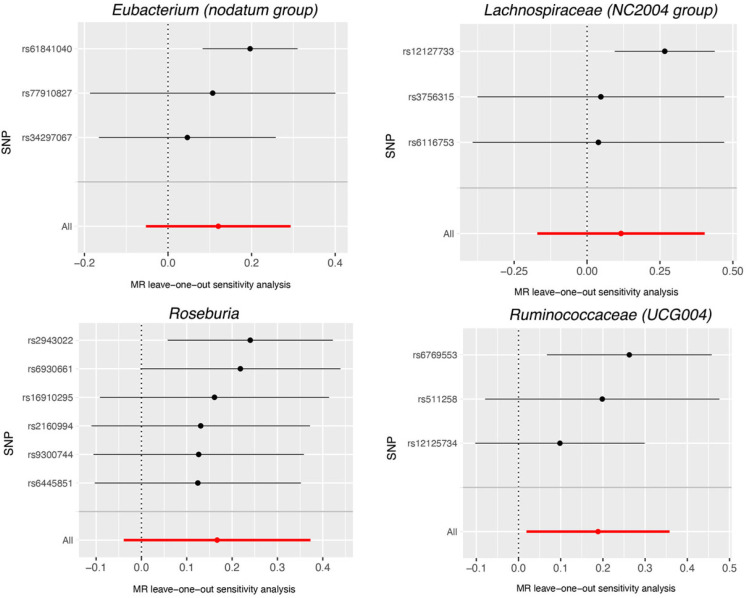
Leave-one-out plots for the causal association between gut microbiota (Eubacterium (nodatum group), Lachnospiraceae (NC2004 group), Roseburia and Ruminococcaceae (UCG004), and glaucoma. The leave-one-out plot presents how the causal estimates (point with horizontal circle) for the effect of gut on glaucoma were influenced by the removal of a single variant. The bars indicate the confidence interval of MR estimates. SNP, single nucleotide polymorphism.

Based on the data provided in Table S5, no significant heterogeneity was detected by Cochran’s IVW Q test. Additionally, the MR-Egger regression intercept analysis, as displayed in Table S6, did not reveal any obvious directional horizontal pleiotropy. The MR-PRESSO method also did not detect any noteworthy exceptional values. Thus, there was insufficient evidence to support the presence of horizontal pleiotropy in the association between these gut microbiota compositions and the two diseases under investigation. To validate the influence of each SNP on the overall causal estimate, a leave-one-out method was employed. Each SNP was systematically removed, followed by the repetition of MR analysis on the remaining SNPs. Figure 4 depicts the leave-one-out plots, illustrating the causal association between gut microbiota (Dorea, Eubacterium (oxidoreducens group), Eubacterium (ventriosum group), Lachnospiraceae (NK4A136 group), Parabacteroides and Ruminococcaceae (UCG009), and AMD. Similarly, Figure 5 illustrates the leave-one-out plots for the causal association between gut microbiota (Eubacterium (nodatum group), Lachnospiraceae (NC2004 group), Roseburia and Ruminococcaceae (UCG004)) and glaucoma. These findings consistently support a significant causal connection among the computed results of all SNPs.

Reverse MR analysis was conducted on the gut microbiota compositions that were identified to have a causal link with AMD and glaucoma in the forward MR analysis. Table 3 lists five MR Methods used to analyze the relationship between AMD and the above gut microbiota. Table 4 lists five MR Methods used to analyze the relationship between glaucoma and the above gut microbiota. The results indicated a correlation between Eubacterium (nodatum group) and glaucoma. As shown in Table 4, the weighted median estimate suggested that glaucoma had a risk effect on Eubacterium (nodatum group) (OR = 1.15, 95% CI, 1.04–1.28, *p* = 0.006). However, we did not find any significant causal association between the two eye diseases and the other mentioned gut microbiota. The Cochran’s IVW Q test results indicated no substantial heterogeneity of these IVs. Furthermore, the results of the MR-Egger regression intercept analysis did not reveal any significant evidence of directional horizontal pleiotropy (Tables S7 and S8).

## 4. Discussion

An MR analysis was conducted in this study using summary statistics data extracted from the IEU Open GWAS Project on gut microbiome, AMD, and glaucoma. The objective was to identify the causal relationship between gut microbiome and AMD/glaucoma. It was found some gut microbiotas (including Eubacterium (oxidoreducens group), Parabacteroides, Lachnospiraceae (NK4A136 group), and Ruminococcaceae (UCG009) that had a significant protective effect on AMD, while Dorea and Eubacterium (ventriosum group) had a risk effect on AMD. Gut microbiotas (including Eubacterium (nodatum group), Lachnospiraceae (NC2004 group), Roseburia, and Ruminococcaceae (UCG004) were all found to have a risk effect on glaucoma. In recent times, mounting evidence has bolstered the understanding of the “gut-eye axis” in the development of several eye disorders [5]. Various cells within the eye, including microglial cells, perivascular macrophages, dendritic cells, and retinal pigment epithelium (RPE) cells express pattern recognition receptors (PRRs) that can be activated by gut-derived cells, thereby provoking ocular inflammation [24]. In addition, microbial byproducts have the potential to trigger autoimmune responses localized within the eye by activating retina-specific signaling pathways [25]. The inseparable link between the gut microbiota and AMD has been reviewed by Lima-Fontes et al. [7]. As for glaucoma, it is a neurodegenerative disease with multifactorial origins, involving inflammation and immune responses in its pathogenesis. Zhang et al. conducted a metagenomic analysis of the gut microbiota in individuals with glaucoma, further confirming the crucial role of the gut microbiota and its derivatives in the initiation and progression of glaucoma [26].

Dorea is the main gas-producing bacterium in the gut. Previous metabolomics analysis has shown that Dorea is involved in the formation of the gut barrier, affects innate immunity, participates in the regulation of the malignant tumor cell cycle, and hosts adaptive immunity [27]. Dorea has been found to be associated with fungal keratitis (FK) [28], Sjögren’s syndrome (SS), and dry eye syndrome (DES) [29]. However, its effects on AMD and glaucoma are rarely reported. Eubacterium, another important genus in the human gut microbiota, is involved in nutrient metabolism and the maintenance of gut homeostasis. It specifically produces short-chain fatty acids (SCFAs), including butyrate, which serves as a vital source of nutrients and energy for the intestinal epithelium. Previous studies have found causal relationships between Eubacterium and diabetic retinopathy (DR) and optic neuritis (ON) [12,14]. In addition, there have been reports of interactions between Eubacterium and AMD, but its correlation with glaucoma has not been reported. Lachnospiraceae and Ruminococcaceae are both core genera of human gut microbiota and may be potentially beneficial bacteria involved in carbohydrate metabolism, producing butyrate as the main source of energy for the host. The increase in their abundance is related to aging [30]. In eye diseases, Lachnospiraceae and Ruminococcaceae are related to bacterial keratitis and uveitis [31,32] and Lachnospiraceae is also related to fungal keratitis and mucous membrane pemphigoid [27,33]. In addition, studies have reported interactions between Lachnospiraceae, Ruminococcaceae, and AMD, but their causal relationship with AMD is still unknown. Their interaction with glaucoma has not been reported yet. Parabacteroides has been less studied in eye diseases, and its effects on AMD and glaucoma are rarely reported. Zysset-Burri et al. [6] compared 57 patients with neovascular AMD to 58 healthy controls in a study investigating the correlation between gut microbiota and AMD. The results indicated that non-AMD patients had significantly higher levels of Oscillibacter and Bacteroides. However, it should be noted that the study did not establish a causal relationship between Oscillibacter or Bacteroides and AMD, possibly due to different types of AMD. Zinkernagel et al. [34] analyzed the gut microbiome of both AMD patients and healthy controls and discovered an increased abundance of Oscillibacter, Anaerotruncus, Eubacterium ventriosum, and Ruminococcus torques in AMD patients. On the other hand, Bacteroides eggerthii was found to be more prevalent in the control group. The study further revealed that Eubacterium ventriosum had a risk effect on AMD. Another study found decreased levels of Oscillospira, Blautia, and Dorea in AMD patients [35]. Notably, this study also found that Dorea had a risk effect on AMD. It was reported that the abundance of Bacteroides and Prevotella is associated with POAG. Gong et al. [8] used 16S rRNA sequencing to detect the fecal microbiota of 30 POAG patients and 30 healthy individuals. Their findings revealed a notable increase in Escherichia coli, as well as unidentified Enterobacteriaceae and Prevotellaceae in POAG patients. Conversely, Bacteroides plebeius and Megamonas were notably reduced. Another study demonstrated a noteworthy reduction in the distribution of Blautia and Fusicatenibacter in fecal samples of patients with primary angle-closure glaucoma (PACG) [9]. However, this study did not establish a significant causal relationship between these bacterial genera and glaucoma, potentially due to variances in the ethnicity of the study participants and disease classification. The above studies mostly focused on Chinese people, while the present study mainly included Europeans.

There are several advantages to this study. By analyzing MR, causal connections between gut microbiota and both AMD and glaucoma can be determined, which eliminates confounding factors and reverses causal inference. We obtained genetic variation data of gut microbiota from the largest GWAS meta-analysis to ensure the reliability of the MR analysis instrument. To detect and eliminate horizontal pleiotropy, we utilized MR-PRESSO and MR-Egger regression interval trial tests. Additionally, we used an MR design and non-overlapping exposure and outcome summary-level data to avoid bias [36]. Nonetheless, this study also has some limitations. Since summary statistics data were used instead of raw data, it is not possible to conduct subgroup analyses, such as distinguishing between early AMD and late AMD, and different types of glaucoma cannot be performed. As the exposure dataset only provides genus-level classification, we are unable to investigate the potential causal link between gut microbiota and AMD/glaucoma at the species level. In order to perform sensitivity analysis and horizontal pleiotropy testing, additional genetic variations must be incorporated as IVs. The sample size for gut microbiota is limited, and instrument bias may weakly influence the results of reverse MR analysis, making it difficult to completely exclude reverse causality. Although most participants in the GWAS gut microbiota data meta-analysis were of European descent, population stratification may still pose a potential confounding factor, rendering the study results not entirely generalizable to non-European populations. To enhance the applicability of deeper investigations into the causal relationships between gut microbiota and AMD/glaucoma, it is recommended to conduct studies in diverse European and non-European populations.

From a clinical practice perspective, the results of this study may contribute to the development of new prevention and treatment strategies for AMD and glaucoma. For example, by adjusting the balance of the gut microbiota, it may be possible to reduce the risk of developing AMD or glaucoma or to improve the severity of these diseases. This can be achieved through changes in diet, supplementation with probiotics, and other methods. However, research in this field is still in its early stages, and more studies are needed to confirm these preliminary findings and to determine the most effective intervention strategies. In addition, the complexity and individual variability of the gut microbiota presents a challenge, which may require personalized treatment plans.

## 5. Conclusions

In conclusion, the present study identified causal relationships between certain gut microbiota including Dorea, Eubacterium (oxidoreducens group), Eubacterium (ventriosum group), Lachnospiraceae (NK4A136 group), Parabacteroides, and Ruminococcaceae (UCG009) with AMD. It also identified a connection between Eubacterium (nodatum group), Lachnospiraceae (NC2004 group), Roseburia, and Ruminococcaceae (UCG004) with glaucoma. The exact protective or risk mechanisms these gut microbiota have on AMD and glaucoma necessitate further Randomized Controlled Trial (RCT) studies. In addition, a reverse MR revealed a causal relationship between glaucoma and Eubacterium (nodatum group) but found no such links between AMD or glaucoma and the other bacterial groups mentioned. Nonetheless, the possibility that AMD or glaucoma could influence the gut microbiota cannot be dismissed, and further research is needed to validate these findings.

## Figures and Tables

**Table 1:** MR estimate for the association between gut microbiota and AMD.

Bacterial Taxa (Exposures)	Methods	SNPs	OR	95% CI	*p*
Dorea	MR Egger	5	1.64	0.64–4.22	0.382
	Weighted median	5	1.50	1.08–2.08	0.016
	IVW	5	1.46	1.15–1.85	0.002
	Simple mode	5	1.55	1.01–2.39	0.116
	Weighted mode	5	1.55	1.04–2.33	0.099
Eubacterium (oxidoreducens group)	MR Egger	4	1.13	0.67–1.90	0.687
	Weighted median	4	0.89	0.72–1.11	0.318
	IVW	4	0.84	0.70–1.00	0.049
	Simple mode	4	0.91	0.68–1.22	0.572
	Weighted mode	4	0.91	0.70–1.18	0.547
Eubacterium (ventriosum group)	MR Egger	8	0.82	0.41–1.66	0.602
	Weighted median	8	1.19	0.92–1.54	0.175
	IVW	8	1.23	1.01–1.50	0.038
	Simple mode	8	1.20	0.82–1.76	0.380
	Weighted mode	8	1.20	0.84–1.73	0.355
Lachnospiraceae (NK4A136 group)	MR Egger	7	0.84	0.63–1.11	0.277
	Weighted median	7	0.81	0.66–0.99	0.041
	IVW	7	0.84	0.71–0.98	0.031
	Simple mode	7	0.79	0.61–1.04	0.143
	Weighted mode	7	0.79	0.63–1.00	0.093
Parabacteroides	MR Egger	3	0.84	0.10–6.75	0.896
	Weighted median	3	0.71	0.48–1.04	0.080
	IVW	3	0.70	0.51–0.96	0.025
	Simple mode	3	0.72	0.45–1.13	0.290
	Weighted mode	3	0.72	0.47–1.11	0.280
Ruminococcaceae (UCG009)	MR Egger	6	0.77	0.21–2.80	0.709
	Weighted median	6	0.76	0.62–0.94	0.011
	IVW	6	0.83	0.70–0.99	0.036
	Simple mode	6	0.72	0.53–0.98	0.093
	Weighted mode	6	0.72	0.52–0.99	0.101

MR, Mendelian randomization; AMD, age-related macular degeneration; SNP, single nucleotide polymorphism; OR, odds ratio; CI, confidence interval; IVM, inverse variance weighted.

**Table 2:** MR estimate for the association between gut microbiota and glaucoma.

Bacterial Taxa (Exposures)	Methods	SNPs	OR	95% CI	*p*
Eubacterium (nodatum group)	MR Egger	3	3.37	0.70–16.2	0.371
	Weighted median	3	1.16	1.01–1.35	0.041
	IVW	3	1.13	0.95–1.34	0.173
	Simple mode	3	1.21	1.01–1.45	0.176
	Weighted mode	3	1.21	1.01–1.45	0.179
Lachnospiraceae (NC2004 group)	MR Egger	3	0.20	0.03–1.19	0.328
	Weighted median	3	1.24	1.03–1.51	0.026
	IVW	3	1.12	0.84–1.50	0.427
	Simple mode	3	1.31	1.01–1.68	0.175
	Weighted mode	3	1.31	1.02–1.68	0.172
Roseburia	MR Egger	6	0.99	0.41–2.41	0.984
	Weighted median	6	1.28	1.03–1.59	0.028
	IVW	6	1.18	0.96–1.45	0.112
	Simple mode	6	1.41	0.98–2.03	0.124
	Weighted mode	6	1.40	0.95–2.05	0.146
Ruminococcaceae (UCG004)	MR Egger	3	1.86	0.93–3.72	0.328
	Weighted median	3	1.17	0.94–1.45	0.161
	IVW	3	1.21	1.02–1.43	0.029
	Simple mode	3	1.14	0.88–1.47	0.482

MR, Mendelian randomization; SNP, single nucleotide polymorphism; OR, odds ratio; CI, confidence interval; IVM, inverse variance weighted.

**Table 3:** MR estimate for the association between AMD and the above gut microbiota.

Bacterial Taxa (Exposures)	Methods	SNPs	OR	95% CI	*p*
Dorea	MR Egger	8	0.96	0.89–1.03	0.274
	Weighted median	8	0.96	0.92–1.01	0.152
	IVW	8	0.96	0.92–1.01	0.098
	Simple mode	8	0.96	0.88–1.04	0.319
	Weighted mode	8	0.96	0.92–1.01	0.191
Eubacterium (oxidoreducens group)	MR Egger	8	1.10	0.96–1.26	0.209
	Weighted median	8	1.02	0.93–1.11	0.713
	IVW	8	1.02	0.95–1.10	0.579
	Simple mode	8	1.05	0.91–1.23	0.518
	Weighted mode	8	1.03	0.95–1.13	0.471
Eubacterium (ventriosum group)	MR Egger	8	1.03	0.95–1.12	0.467
	Weighted median	8	1.03	0.97–1.09	0.330
	IVW	8	1.03	0.99–1.08	0.163
	Simple mode	8	0.99	0.90–1.08	0.807
	Weighted mode	8	1.02	0.97–1.08	0.488
Lachnospiraceae (NK4A136 group)	MR Egger	8	0.93	0.87–1.01	0.124
	Weighted median	8	0.96	0.91–1.01	0.114
	IVW	8	0.96	0.92–1.01	0.086
	Simple mode	8	1.00	0.92–1.09	0.970
	Weighted mode	8	0.95	0.91–1.00	0.103
Parabacteroides	MR Egger	8	0.94	0.85–1.04	0.302
	Weighted median	8	0.98	0.94–1.03	0.483
	IVW	8	1.00	0.94–1.06	0.990
	Simple mode	8	0.96	0.88–1.04	0.307
	Weighted mode	8	0.98	0.93–1.03	0.419
Ruminococcaceae (UCG009)	MR Egger	8	1.00	0.97–1.16	0.964
	Weighted median	8	1.03	0.95–1.12	0.486
	IVW	8	1.04	0.96–1.12	0.308
	Simple mode	8	0.95	0.83–1.08	0.430

MR, Mendelian randomization; AMD, age-related macular degeneration; SNP, single nucleotide polymorphism; OR, odds ratio; CI, confidence interval; IVM, inverse variance weighted.

**Table 4:** MR estimate for the association between glaucoma and the above gut microbiota.

Bacterial Taxa (Exposures)	Methods	SNPs	OR	95% CI	*p*
Eubacterium (nodatum group)	MR Egger	81	1.06	0.87–1.28	0.578
	Weighted median	81	1.15	1.04–1.28	0.005
	IVW	81	1.07	1.00–1.14	0.052
	Simple mode	81	1.27	0.97–1.67	0.086
	Weighted mode	81	1.24	0.99–1.55	0.071
Lachnospiraceae (NC2004 group)	MR Egger	75	0.89	0.77–1.03	0.113
	Weighted median	75	0.95	0.88–1.03	0.217
	IVW	75	1.01	0.95–1.06	0.845
	Simple mode	75	0.93	0.78–1.11	0.433
	Weighted mode	75	0.92	0.81–1.06	0.263
Roseburia	MR Egger	84	0.96	0.89–1.04	0.309
	Weighted median	84	1.00	0.96–1.05	0.906
	IVW	84	1.00	0.97–1.03	0.996
	Simple mode	84	0.95	0.87–1.03	0.236
	Weighted mode	84	0.96	0.90–1.03	0.293
Ruminococcaceae (UCG004)	MR Egger	83	0.99	0.89–1.11	0.906
	Weighted median	83	1.05	0.99–1.12	0.113
	IVW	83	1.00	0.96–1.04	0.886
	Simple mode	83	1.08	0.93–1.25	0.322

MR, Mendelian randomization; SNP, single nucleotide polymorphism; OR, odds ratio; CI, confidence interval; IVM, inverse variance weighted.

## Data Availability

Data are available on reasonable request.

## Supplementary Materials

The following supporting information can be downloaded at: https://www.mdpi.com/article/10.3390/nu15214646/s1, Table S1: SNPs information of bacteria with AMD; Table S2: SNPs information of bacteria with glaucoma; Table S3: MR estimates of bacteria on AMD; Table S4: MR estimates of bacteria on glaucoma. Table S5: Heterogeneity tests of bacteria on AMD and glaucoma; Table S6: Horizontal pleiotropy tests of bacteria on AMD and glaucoma; Table S7: Heterogeneity and horizontal pleiotropy tests of AMD on bacteria. Table S8: Heterogeneity and horizontal pleiotropy tests of glaucoma on bacteria.
